# IgA Nephropathy: Epidemiology, Outcomes, and Insights for Primary Glomerulonephritides

**DOI:** 10.3390/jcm15052046

**Published:** 2026-03-07

**Authors:** Zuzanna Jakubowska, Filip Wantoch-Rekowski, Jacek S. Małyszko, Jolanta Małyszko

**Affiliations:** 1 Department of Nephrology, Dialysis and Internal Medicine, Medical University of Warsaw, 02-097 Warsaw, Poland; zuzanna.jakubowska@wum.edu.pl (Z.J.); filip.rekowski@uckwum.pl (F.W.-R.); 2 1st Department of Nephrology, Transplantology and Internal Medicine with Dialysis Unit, Medical University of Bialystok, 15-540 Bialystok, Poland; jackmaly@poczta.onet.pl

**Keywords:** chronic kidney disease, glomerulonephritis, IgA nephropathy, epidemiology, translational, kidney transplantation, recurrence

## Abstract

According to the Global Burden of Disease 2019 analysis, there were 606,300 new cases of chronic kidney disease due to glomerulonephritis worldwide, with 17.3 million prevalent cases and 183,700 deaths More interestingly, between 1990 and 2019, the global burden of glomerulonephritis increased by 77% in incidence and 81% in prevalence, mainly due to demographic aging and population growth. Among primary glomerulopathies, IgA Nephropathy (IgAN), also known as Berger’s disease, is the most common primary glomerulopathy worldwide, with significant geographic and ethnic variation in incidence, with the highest prevalence in Europe and Asia and the lowest in Africa. Its pathogenesis reflects a complex interaction between polygenic susceptibility and environmental modifiers, mucosal immune activation, infections of the upper respiratory and gastrointestinal tracts, dietary factors, and alterations in the gut microbiome. In addition, IgAN increasingly coexists with other chronic diseases, such as hypertension and diabetes, which complicates both diagnosis and treatment in aging societies. All these observations suggest that in the coming years, the epidemiology of IgAN will gradually transform from a description of “case counts” to a predictive tool that integrates genetic, environmental, and molecular biomarker data. In this sense, epidemiology is increasingly becoming the foundation of precision nephrology—allowing not only for disease risk prediction but also for the design of effective therapeutic strategies. The conceptual shift in IgAN—from a disease defined by biopsy prevalence to one understood through integrative epidemiology—illustrates the broader transition of GN research toward biomarker-based risk stratification and precision medicine. This review focuses on IgA nephropathy as the most prevalent primary glomerulonephritis and uses it as a reference disease to illustrate broader epidemiological patterns, outcome trajectories, and methodological limitations relevant to primary glomerulonephritides.

## 1. Introduction

A foundational step towards developing effective strategies to improve health in the general population is to assess causes of death. The Global Burden of Diseases, Injuries, and Risk Factors (GBD) study provides comprehensive and systematic analyses of mortality causes worldwide and across time [1,2]. In addition, the GBD study makes all the data freely available and is updated and based on the scientific literature published. Moreover, the GBD study also further explores the relationship between age and cause of mortality. The GBD study also provides researchers, policymakers, stakeholders, and the general public with tools based on evidence to better understand the global impact of injuries, diseases, and modifiable risk factors at the population level [3]. No primary data analysis was performed, and all epidemiological estimates are cited from original GBD publications and registries. In addition, we would like to emphasize the GBD uncertainty intervals and modeling assumptions [4].

Key model assumptions of the framework include comprehensiveness and consistency, data synthesis (with sparsity of direct data; statistical methods such as DisMod-MR is used by the GBD study to “borrow strength” from related locations, ages, or time periods), uncertainty intervals, risk-attributable burden, iterative-re-estimation (every year the entire time series is re-estimated to incorporate new data to ensure longitudinal consistency), and the methodological framework (with key inputs such as death, surveillance, survey and hospital data, with covariates like education to model trends). In the GBD study, every estimate is calculated 1000 times, and the 95th uncertainty interval is determined by the 25th and 975th values out of 1000 values presented in order from the smallest to the largest from a 250-draw distribution. The former results from extensive datasets, large studies, and consistent data across sources. On the other hand, the latter results from limited data availability, small sample sizes, and inconsistent, conflicting data.

As observed in nephrology, in recent years, many new therapies emerged, with most mainly involving the glomerulonephritis and vasculitis; therefore, this scoping review was conducted according to the PRISMA-ScR (Preferred Reporting Items for Systematic Reviews and Meta-Analyses extension for Scoping Reviews) guideline to map the epidemiology of primary glomerulonephritides (GNs), mainly IgA nephropathy; characterize regional and temporal trends; and summarize translational implications for clinical practice.

## 2. Methods

**Figure 1:**
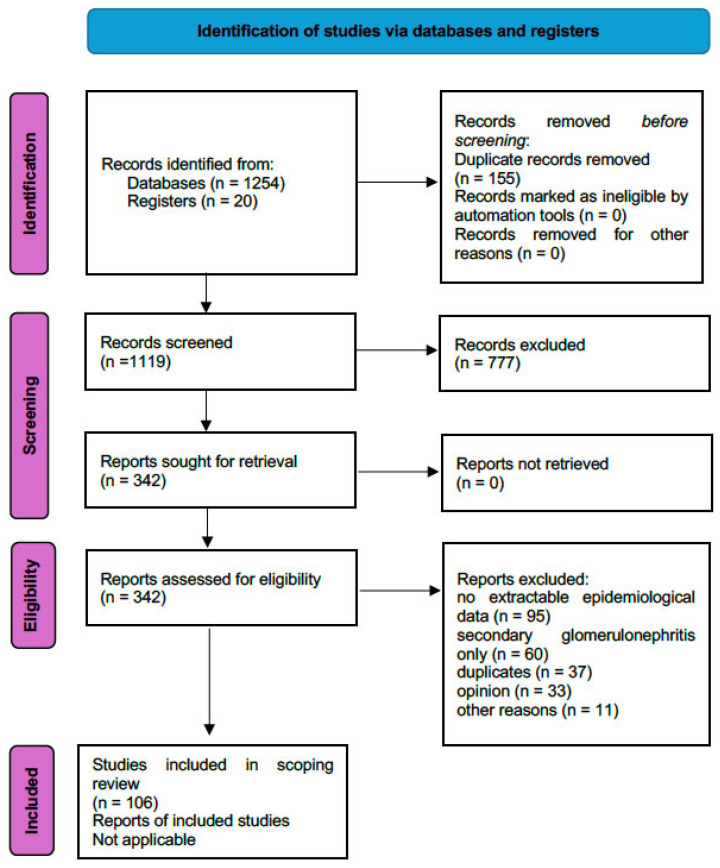
PRISMA 2020 flow diagram for new systematic reviews, which included searches of databases and registries only.

A review protocol was developed a priori and defined the objectives, eligibility criteria, search strategy, and data extraction framework. Our team included population-based studies; regional registry analyses; large cohort studies reporting the incidence, prevalence, geographic distribution, or temporal trends of primary GNs; systematic reviews and meta-analyses on GN epidemiology; and studies linking epidemiological characteristics with clinical outcomes or management implications. We excluded case reports, studies without extractable epidemiological data, reports focusing exclusively on secondary GNs (e.g., diabetes and amyloidosis), and conference abstracts. The population covered by the review was people with biopsy-proven or clinically diagnosed primary glomerulonephritis, including IgA nephropathy (IgAN), membranous nephropathy (MN), focal segmental glomerulosclerosis (FSGS), minimal change disease (MCD), membranoproliferative GN/C3 glomerulopathy (MPGN/C3G), and ANCA-associated GNs. A systematic search was conducted by 4 independent authors in PubMed/MEDLINE and supplemented by hand-searching the reference lists of key reviews and major nephrology guidelines (KDIGO and ERA) (Figure 1). We confirm compliance with PRISMA, which was not registered. The completed PRISMA-ScR checklist [5] is available in the Supplementary Materials.

The literature search was conducted in PubMed/MEDLINE and covered publications from January 2000 to 28 November 2025, using predefined disease-specific terms for primary glomerulonephritis (including IgA nephropathy, membranous nephropathy, focal segmental glomerulosclerosis, minimal change disease, membranoproliferative glomerulonephritis/C3 glomerulopathy, and ANCA-associated glomerulonephritis) combined with epidemiology-related keywords (epidemiology, incidence, prevalence, registry, population-based, and global burden), and it was restricted to English-language studies in humans. Full texts were assessed when eligibility could not be determined from the abstract. Discrepancies were resolved by discussion. Due to heterogeneity in study designs, definitions of GNs, biopsy practices, and reporting metrics, a quantitative synthesis was not appropriate. Findings were therefore summarized narratively and organized into: global epidemiological overviews, regional trends, IgAN-specific epidemiology, epidemiology of other primary GNs, and translational implications for clinical practice. Methodological limitations of the included studies—such as biopsy-based selection bias, inconsistent diagnostic criteria, and underrepresentation of certain regions—were qualitatively evaluated and summarized.

## 3. IgAN as a Paradigm

IgA nephropathy (IgAN) uniquely combines four features central to primary glomerulonephritis epidemiology: marked geographic variability, dependence on biopsy-based detection, highly heterogeneous clinical trajectories, and emerging biomarker-based risk stratification [6,7,8,9,10].

Unlike several other primary glomerulonephritides that are regionally restricted or age-specific, IgAN occurs across all continents and age groups while demonstrating substantial variation in reported incidence and prevalence driven by differences in biopsy practices, screening strategies, and healthcare access [6,8,11,12,13]. This makes IgAN particularly suitable as a reference condition for examining how diagnostic intensity and ascertainment bias shape observed epidemiology across glomerular diseases. Furthermore, IgAN illustrates the limitations of traditional clinicopathologic classification systems. Long-term cohort studies show that patients with similar histologic patterns and baseline clinical characteristics may experience widely divergent renal trajectories, highlighting the need for multivariable prognostic models and dynamic risk assessment approaches that are increasingly applied across primary glomerulonephritides [10,14,15]. IgAN is also among the first glomerular diseases in which epidemiology, genetics, and molecular biomarkers were integrated into predictive tools and targeted therapeutic strategies, as reflected in international risk-prediction models and emerging pathway-directed therapies [13,16,17]. This transition from descriptive epidemiology toward precision nephrology is likely to extend across the broader spectrum of primary glomerulonephritides.

For these reasons, IgAN can be considered a reference and a paradigmatic disease within primary glomerulonephritis, providing a structured lens through which broader epidemiological patterns, methodological constraints, and translational challenges in glomerular disease research can be examined.

## 4. Global Epidemiology—Regional Overview

According to the Global Burden of Disease 2019 analysis, there were 606,300 new cases of chronic kidney disease due to glomerulonephritis worldwide, with 17.3 million prevalent cases and 183,700 deaths [1]. The estimated global incidence rates for primary glomerulonephritis types are: IgA nephropathy: 2.5 per 100,000 people per year, membranous nephropathy: 1.2 per 100,000 people per year, focal segmental glomerulosclerosis: 0.8 per 100,000 people per year, and minimal change disease: 0.6 per 100,000 people per year.

**Figure 2:**
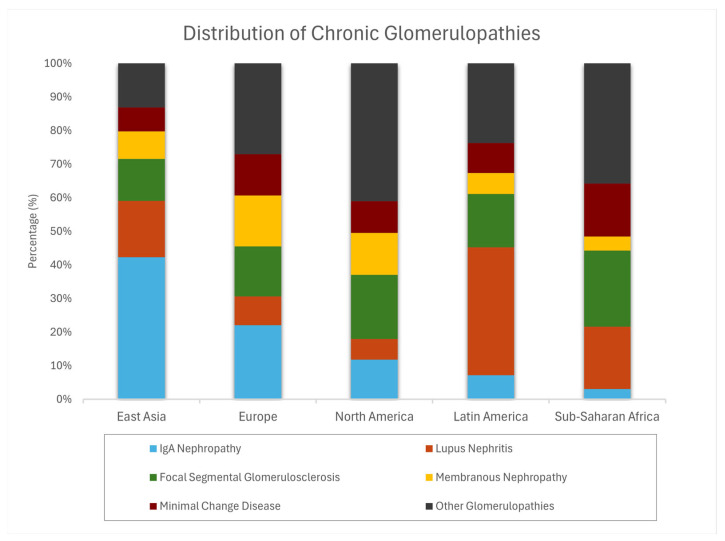
Global distribution of chronic glomerulonephritis.

Between 1990 and 2019, the global burden of glomerulonephritis increased by 77% in incidence and 81% in prevalence, driven primarily by demographic aging and population growth [1]. A multicontinental analysis of 29 nephropathology laboratories demonstrated marked regional heterogeneity in glomerular disease distribution. In North America, diabetic glomerulosclerosis and FSGS each accounted for 19.1% of biopsies; Latin America was characterized by a predominance of lupus nephritis (38.1%) and FSGS (15.8%); European cohorts were dominated by IgA nephropathy (22.1%) and FSGS (14.9%); and Asian centers reported IgA nephropathy (39.5%) and lupus nephritis (16.8%) as the most frequent diagnoses [6]. The global distribution of chronic glomerulonephritis is presented in Figure 2.

## 5. Epidemiology of Primary Glomerulonephritis (Non-IgA)

**Figure 3:**
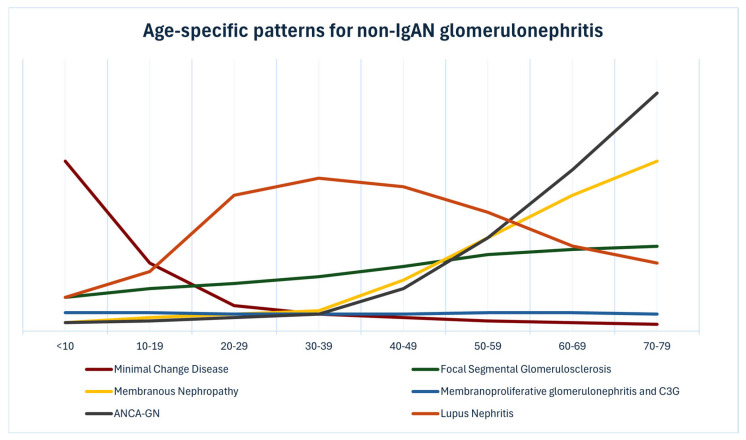
Age-specific pattern of glomerulonephritis other than IgA nephropathy.

Primary glomerular diseases include minimal change disease (MCD), focal segmental glomerulosclerosis (FSGS), membranous nephropathy (MN), membranoproliferative glomerulonephritis/C3 glomerulopathy (MPGN/C3G), and ANCA-associated glomerulonephritis (ANCA-GN) [18]. The epidemiology of these conditions is complex and geographically variable. In adults, individual disease incidence broadly ranges from 0.1 to 1.2 per 100,000 person-years, with substantial variation depending on geographic location and ethnicity [19]. The age-specific pattern of glomerulonephritis other than IgA nephropathy is given in Figure 3.

Membranous nephropathy (MN) deserves particular attention as a major contributor to nephrotic syndrome in older populations [20]. The reported worldwide incidence approximates 1.2 per 100,000 persons annually—making MN one of the leading causes of severe nephrotic syndrome, particularly in the aging population [21].

Over the past several decades, a notable shift was observed in the prevalence and incidence rates of focal segmental glomerulosclerosis (FSGS). A recent study from the US reported an increasing prevalence of FSGS between 2016 and 2020, particularly among Black individuals [22]. The average annualized and standardized prevalence was 212.6 per million, which increased every year, starting at 158 per million in 2016 to 260.1 per million in 2020 [11]. A national 30-year Danish study [23] confirmed a marked increase in FSGS incidence from 1.5 to 5.7 per million person-years between 1985 and 2014, validating the claim of substantial FSGS increases over recent decades.

For ANCA-associated vasculitis (AAV), a systematic review and meta-analysis of 25 studies found a global pooled incidence of 17.2 per million person-years (95% CI: 13.3–21.6) with a prevalence of 198 per million persons [24]. A Swedish 23-year study documented stable AAV incidence at approximately 30 per million adults, with its prevalence rising to 428 per million [25].

MCD dominates in children and is the leading cause of Idiopathic Nephrotic Syndrome (INS), accounting for 70–90% of cases [26]. INS incidence serves as a proxy for MCD trends and shows marked ethnic disparity, with incidence rates showing high ethnic disparity: approximately 2.0 per 100,000 person-years in Caucasian children compared to 6.2–15.6 per 100,000 in Asian populations and 9.2 per 100,000 in Arabian children. In adults, MCD is rarer (0.2 per 100,000) and less dependent on ethnicity [27,28].

Membranoproliferative glomerulonephritis (MPGN) and complement 3 glomerulopathy (C3G) remain uncommon even within glomerulonephritis diagnoses. MPGN and C3G are characterized by thickening of capillary walls and mesangial proliferation with the immune complex and complement deposits. The risk of progression to advanced kidney disease is very high [23,29]. In recent studies, new emerging complement inhibitors such as Iptacopan or Pegcetacoplan demonstrated a significant reduction in proteinuria and stabilization of kidney function [30,31]. For MPGN/C3G, the stated incidence is 0.1–0.2 per 100,000.

Although the age-standardized rates of glomerulonephritis have declined in recent decades—reflecting improved treatment and management—overall prevalence and absolute case numbers are projected to rise significantly by 2040. This reflects two opposing forces: better treatment outcomes due to global population growth and the aging demographic structure worldwide [32]. Its epidemiology, temporal trend, and ESRD risk of various glomerulonephritis are given in Table 1.

## 6. Epidemiology of IgA Nephropathy (IgAN)—Significance, Conditions, and Prognosis

This section summarizes the epidemiology, clinical course, and translational implications of IgA nephropathy.

### 6.1. Regional Distribution

IgA nephropathy (IgAN) demonstrates pronounced geographic variability in incidence and biopsy prevalence worldwide. The highest frequencies are consistently reported in the Asia–Pacific region, where IgAN constitutes a substantial proportion of biopsy-proven primary glomerulonephritis and annual incidence often exceeds 3–4 per 100,000 population [19,33,34]. Within Asia, prevalence is generally highest in East Asian countries and lower in those in South and Southeast Asia, while Australia represents a lower-incidence outlier [19,33,34].

European cohorts report intermediate IgAN frequencies, typically around one-fifth to one-quarter of biopsy-proven glomerulonephritis, with incidence generally between 1 and 3 per 100,000 population [6,11,35]. Considerable variability exists between countries and registries, partly reflecting differences in biopsy rates and healthcare access [11,36].

In North and Latin America, IgAN represents a smaller proportion of glomerulonephritis compared with Europe and Asia. The reported incidence is generally around 1–1.5 per 100,000 population, and registry data indicate lower biopsy prevalence relative to other primary glomerular diseases, particularly in regions where lupus nephritis predominates [6,19].

Sub-Saharan Africa shows the lowest reported IgAN frequencies globally, often below 5% of glomerulonephritis diagnoses. Limited biopsy availability, restricted nephropathology infrastructure, and under-ascertainment are considered major contributors to these low estimates rather than the true absence of disease [6,19].

Detailed data are presented in Table 2.

### 6.2. Prevalence in Populations

**Figure 4:**
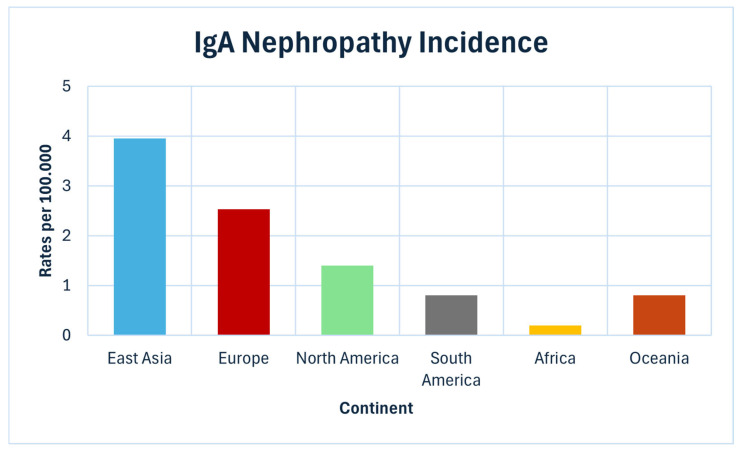
Incidence of IgA nephropathy by continent.

Epidemiological data clearly confirm that IgAN occurs with varying prevalence rate depending on the geographic region (Figure 4). In East Asian countries, such as Japan and China, IgAN accounts for 30–40% of all primary glomerulopathies detected in renal biopsies. In contrast, in Europe, it accounts for approximately 20%, and in North America, it only accounts for 10% of cases [7]. In Japan, the incidence is estimated at 3.9–4.5 per 100,000 persons per year [49], while in the United States, it is approximately 1.3–2.2 per 100,000 person-years (standardized 1.4/100,000) [9]. In Africa, IgAN is diagnosed significantly less frequently, which may be due in part to the limited availability of renal biopsies and different genetic predispositions [8]. It is worth emphasizing that local clinical practices significantly influence epidemiology. Countries such as Japan and South Korea conduct population-based screening programs (including urine tests in children and adolescents), which allow for the detection of IgAN in its early stages and increase disease reporting [50]. In European countries and North America, where biopsy is primarily directed at patients with proteinuria and impaired renal function, IgAN is usually diagnosed later, which is associated with a poorer prognosis [12].

In the recent systematic review with 39 studies included [51], the authors aimed to integrate data from both regional and national registries of kidney biopsy to evaluate the global distribution of biopsy-proven glomerular diseases. They found that the most common primary glomerulonephritis worldwide was IgAN, with exceptionally high prevalence in East Asia (up to 35.8%) and moderate prevalence in Europe (up to 20%) [51]. They stressed the critical role of kidney biopsy registries in research. They also advocate international harmonization of kidney biopsy protocols as well as diagnostic standards. These conclusions are further in line with the previous review by Schena and Nistor on the epidemiology of IgAN published in 2018 [52]. They underlined the role of geographical variation in data interpretation, as the incidence and clinical patterns of IgAN differ markedly across regions and may also influence disease presentation and prognosis in elderly populations.

In addition, Ghaddar et al. [53] in another recent review also considered geographic variation in IgAN incidence with an increase from west to east and south to north across Eurasia to be partly explained by the distribution of genetic risk alleles in individuals of European and East Asian ancestry. They also stressed that epidemiological studies with an appropriate and well-defined population and infrastructure are limited. As the diagnosis of IgAN is based on the kidney biopsy, the incidence of IgAN is subject to bias due to access to healthcare, including nephropathology, referral patterns, screening programs, and appropriate financial resources. It also highlights the need for public health efforts tailored to the changing spectrum of kidney diseases, including rare diseases such as primary glomerulonephritides.

### 6.3. Genetic and Environmental Factors

IgAN is a disease with a clear polygenic basis. Analyses of family studies and GWAS (genome-wide association studies) indicate a significant association with specific HLA alleles (HLA-DRB1 and HLA-DQB1), as well as with HLA-independent regions such as CCR6, STAT3, and CFB, particularly in the Han Chinese population [54]. Additionally, a low copy number of the DEFA1A3 gene, which encodes α-defensins, is associated with a higher risk of disease onset and progression [55].

The environment plays a role in the unfolding of genetic predispositions. Upper respiratory and gastrointestinal infections often precede episodes of hematuria, suggesting that activation of mucosal immunity is a key pathogenetic mechanism [7]. Changes in the gut microbiome and diet may also modulate disease risk, although evidence in this area is still limited. Lifestyle factors such as smoking and low physical activity have also been associated with faster progression of IgAN [16].

### 6.4. Epidemiology and Prognosis

The epidemiological dimension directly impacts the prognosis of patients with IgAN. In populations where diagnostics are more intensive, the disease is detected at earlier stages, offering a greater chance of effectively slowing its progression. It is estimated that 10–20% of patients develop end-stage renal disease (ESKD) within 10–20 years of diagnosis, but this risk strongly depends on clinical (proteinuria and eGFR), histopathological (MEST-C classification), and genetic factors [15,16]. New prognostic models integrating this data, such as the international IgAN Prediction Tool, allow for increasingly better risk stratification and personalized treatment [16,56]

### 6.5. Changing Trends and Future Prognosis

Several distinct trends in the epidemiology of IgAN have been observed in recent decades. In many regions, the number of diagnoses has increased, but this has largely been due to expanded screening and improved availability of kidney biopsies and not necessarily due to an increase in the actual incidence rate [19]. At the same time, thanks to screening tests, diagnoses are increasingly being made in earlier stages of the disease, which has a positive impact on prognosis and changes the natural course of IgAN [12]. Additionally, environmental changes, such as improved hygiene or dietary modifications, may in the future influence the risk of disease occurrence and its clinical course [57].

Equally important is the fact that in aging societies, IgAN increasingly coexists with other chronic diseases, such as hypertension and diabetes, which complicates both diagnosis and treatment [58]. In recent years, the outlook for patients with IgAN has significantly improved thanks to the introduction of new, targeted therapies. Drugs that modulate the immune response in the mucosa, such as oral budesonide with local release in the distal intestine or complement inhibitors (e.g., iptacopan and narsoplimab), offer a real chance to slow disease progression and reduce the risk of developing end-stage renal disease [17]. The results of recent clinical trials indicate that these therapies effectively reduce proteinuria and stabilize the glomerular filtration rate, which in the long term may change the natural course of IgAN [59].

All these observations suggest that in the coming years, the epidemiology of IgAN will gradually transform from a description of “case counts” to a predictive tool that integrates genetic, environmental, and molecular biomarker data. In this sense, epidemiology is becoming the foundation of precision medicine in nephrology—allowing not only for disease risk prediction but also for the design of effective therapeutic strategies. These epidemiological patterns collectively reinforce the role of IgA nephropathy as a reference disease, illustrating how diagnostic practices, population structure, and therapeutic advances shape the observed glomerulonephritis epidemiology.

### 6.6. Molecular Basis of IgA Nephropathy

IgA nephropathy is driven by dysregulation of mucosal IgA immune responses and is classically explained by the four-hit hypothesis. Disease development involves increased production of galactose-deficient IgA1 (Gd-IgA1), formation of antiglycan autoantibodies, circulating immune-complex generation, and mesangial deposition leading to glomerular injury [8,60]. Genetic susceptibility influences several steps of this process, including variants affecting IgA1 O-glycosylation pathways such as C1GALT1, which may contribute to ethnic differences in circulating Gd-IgA1 levels and disease epidemiology [8,60].

Mucosal immune activation is a central upstream driver. Cytokines regulating B-cell maturation and IgA production, particularly BAFF and APRIL, promote generation of Gd-IgA1-secreting plasma cells and represent emerging therapeutic targets [61,62]. Environmental modifiers, including mucosal dysbiosis, may further influence abnormal IgA production and immune-complex formation [63,64].

Downstream injury results from mesangial deposition of Gd-IgA1-containing immune complexes and activation of complement pathways, leading to cellular proliferation and progressive kidney damage [7]. Integration of molecular pathways with clinical and biomarker-based risk stratification is increasingly redefining IgAN from a purely biopsy-defined disease toward a molecularly characterized glomerulopathy, reflecting the broader transition of glomerulonephritis research toward precision-based classification and targeted therapy [7,8].

### 6.7. Progression to End-Stage Kidney Disease

New KDIGO recommendations from 2025 [13] aimed to reduce proteinuria to <0.5 g/day, ideally <0.3 g/day to achieve complete remission, while older KDIGO guidelines recommended reducing proteinuria to <1 g/day [50]. These new recommendations incorporated data from recent large European cohort studies reporting that even lower levels of proteinuria or albuminuria were also associated with a significant risk for long-term kidney failure [36,65,66]. Rahmani et al. [67] analyzed the IgA nephropathy cohort within the European Rare Kidney Disease Registry (ERKReg) (884 adults and 385 children with a biopsy-proven diagnosis of IgAN from 10 European countries and 49 units) and compared it to the subgroup from the RaDaR study [68] that was composed of patients with proteinuria > 0.5 g/d or eGFR < 60 mL/min/1.73 m^2^ at any time. They found that in this large European IgA nephropathy cohort, adult presentation of IgAN and greater time-averaged proteinuria led to poorer kidney survival. More interestingly, the resolution of microhematuria was associated with more favorable outcomes [69]. Therefore, eradicating hematuria, in addition to a reduction in proteinuria to less than 0.3 g/day, may become the new target in the therapy of IgAN [70]. In Romania, elderly patients (≥60 years with biopsy-proven IgAN) diagnosed between 2010 and 2024 at a tertiary center in Bucharest were followed for over a median follow-up period of 5 years, and almost half of them (48%) reached the composite outcome (ESKD—defined as initiation of kidney replacement therapy (hemodialysis, peritoneal dialysis, or kidney transplantation)—or death before the start of kidney replacement therapy), including 32 (31%) who progressed to ESKD [71].

Additionally, recent clinical trials observed the disappearance of microhematuria with newer immunomodulatory therapies, suggesting that resolution of hematuria could become an achievable therapeutic goal in IgAN management [70,72].

## 7. Comparative Epidemiology and Outcomes Across Primary Glomerulonephritides: Insights from IgA Nephropathy

IgA nephropathy, as outlined in Section 3, provides a reference framework for interpreting epidemiological heterogeneity and prognostic variability across primary glomerulonephritides. As the most prevalent and globally distributed form of primary glomerulonephritis, IgAN provides a clinically grounded reference for comparing epidemiological gradients and outcome trajectories across GN subtypes. Comparative analysis of the major primary glomerulonephritides indicates that similarities in glomerular damage location do not translate into uniform clinical trajectories or long-term prognosis. IgA nephropathy, focal segmental glomerulosclerosis, membranous nephropathy, and minimal change disease differ significantly in terms of age of onset, predominant clinical presentation, rate of renal function loss, and risk of progression to end-stage renal disease. Data from large population-based registries and long-term cohort studies suggest distinct prognostic patterns depending on etiology [23]. In particular, IgA nephropathy and focal segmental glomerulosclerosis are characterized by a relatively more rapid decline in glomerular filtration rate and a higher risk of requiring renal replacement therapy compared to membranous nephropathy and minimal change disease, which more often exhibit a slower course and a more favorable long-term renal prognosis. At the same time, patients with primary glomerulonephritis—regardless of subtype—often have a lower risk of cardiovascular events and all-cause mortality than patients with non-immune chronic kidney disease, highlighting the distinctiveness of this disease group within the CKD population [73]. An important finding from interindividual comparisons is the observed inconsistency between classical histopathological categories and the actual clinical course of the disease. Long-term studies show that patients with similar baseline characteristics can exhibit dramatically different trajectories of renal function loss, limiting the predictive value of single-item clinical and histological assessments and necessitating a dynamic, multi-item approach to risk assessment [14]. A summary of key epidemiological and prognostic features of the main primary glomerulonephritis diseases is presented in Table 3, which synthesizes available registry and cohort data and illustrates both common features of this group of diseases and significant differences in clinical and epidemiological significance.

## 8. IgA Nephropathy (IgAN)—Special Populations

### 8.1. Gender Differences and the Context of Pregnancy in IgA Nephropathy

One area that is under-recognized in IgAN research is gender differences and the influence of hormonal status on the course of the disease. Although IgA nephropathy is more frequently diagnosed in men, available epidemiological and clinical data indicate that women may experience a different progression and respond differently to risk factors, suggesting the potential involvement of hormonal and immunological mechanisms in modulating disease activity [53,74]. This variability becomes particularly important in the context of pregnancy, which is associated with profound remodeling of the immune response and a physiological increase in renal hemodynamic load. Evidence regarding the effects of IgAN on pregnancy outcomes is limited and confusing. In the study by Limardo et al. [75], it was shown that pregnancy in women with IgAN and preserved kidney function did not seem to affect the long-term outcome of kidney disease. In addition, in this population, changes in proteinuria during a median follow-up of 10 years or the risk of new-onset hypertension were not affected by pregnancy. On the other hand, in a register-based cohort study in a nationwide cohort of women with biopsy-verified IgA nephropathy in Sweden, Jarrick et al. [76] found that this type of glomerulonephritis was significantly related to risks of preterm birth, small-for-gestational-age birth, preeclampsia, and cesarean section. On the other hand, the absolute risk of intrauterine and neonatal death was small and similar to that of the reference population of matched women without IgAN. In the Japanese case–control study using data from 924,238 patients with CKD obtained from a hospital claims database, including 297 pregnancies with IgAN, use of antihypertensive medication pre-conception was considered as a surrogate marker for underlying hypertension [77]. They found that pregnant females on drugs other than renin–angiotensin–aldosterone system inhibitors had a significantly higher risk of preterm delivery when compared with those on only renin–angiotensin–aldosterone system inhibitors. Liu et al. [78] reported in a systematic review that the risk for adverse renal outcomes was not increased in predominantly early IgAN. On the other hand, Piccoli et al. [79] in their systematic review suggested that pregnancy in females with IgAN is associated with an increased risk of preeclampsia, preterm birth, and low-for-gestational-age birth weight. In the most recent studies, cases of worsening proteinuria, worsening renal function, and an increased risk of obstetric complications have also been reported, especially in patients with a pre-existing active disease or impaired renal function prior to conception [14,73]. However, interpretation of available data remains limited because pregnant women were often excluded from observational studies, glomerular disease registries, and prognostic studies, leading to underrepresentation of this population and hindering the formulation of clear clinical conclusions [33]. This gap highlights the need to design future population-based and registry studies that incorporate gender and pregnancy status as prognostically significant variables, thereby further reinforcing the role of IgA nephropathy as a paradigmatic disease and revealing the limitations of current risk stratification models.

### 8.2. IgA Vasculitis, Formerly Henoch–Schönlein Purpura

English physician William Heberden described for the first time two boys with clinical findings of purpuric rash, arthralgia, and abdominal pain [80]. Henoch–Schönlein purpura was named after two 19th century German physicians, Johann Schönlein and his student Eduard Henoch. The first case, which was described in 1837, described the coincidence of non-thrombocytopenic purpura with joint pain and called it purpura rheumatica [81], while the second one, which was added in 1874, indicated involvement of the gut and kidney [82]. Now the preferred term is IgA vasculitis (IgAV), and it is defined as complex immune-mediated vasculitis with small blood vessel involvement in various organ systems [83]. Small vessels of the joints, kidneys, gastrointestinal tract, and skin are the most affected, while the central nervous system and lungs are seldom involved. It should be stressed that many overlapping features exist between IgAV with renal involvement and IgAN. Of note, children younger than 15 years of age are more likely to suffer from IgAV with nephritis, whereas in older children and adults, IgAN is more prevalent. Clinical presentation of IgAV includes systemic vasculitis with predominantly extrarenal involvement such as palpable purpura, arthritis/arthralgia, and gastrointestinal symptoms following a preceding infection (mainly from the upper respiratory tract, which occurs less often after antecedent gastrointestinal infection or pharyngitis), whereas IgAN commonly presents with hematuria [84]. Boys have a slightly higher incidence of IgA than girls, whereas in adults, there is equal predilection [85]. An association with the HLA-DQA1 and DQB1 intergenic zone, the HLA-DRB1*01:11/B1*13 loci, and the DQA1*01:01/DQB1*05:01/DRB1*01:01 haplotype was reported [86]. IgAV with renal involvement is more often diagnosed during the winter season, whereas IgAN does not exhibit seasonality [83]. IgAV pathophysiology is not fully understood; however, a significant role appears to be played by IgA [83]. The most definitive tool to diagnose both IgAV with kidney involvement and IgAN is renal biopsy. In the former, capillary and subendothelial IgA deposits and neutrophilic infiltration are likely to be present [84,87]; moreover, IgA-mediated immune-complex deposits may cause mild proliferative or severe crescentic glomerulonephritis [84,87]. In addition, mixed immunocomplexes with IgA and IgG components could be found in IgAV, while only IgA complexes are found in IgAN. Last but not least, the vast majority of IgAV with nephritis end up in clinical remission, as the extent of renal involvement defines long-term morbidity [83]. Kidney transplantation will be required in approximately 1% of children with IgAV [88], whereas IgAN progresses to end-stage kidney disease, requiring renal replacement therapy within 20 years of diagnosis in 30% to 50% of cases [84].

### 8.3. Recurrence After Transplantation

Histologic recurrence of glomerulonephritis, with or without evidence of clinical disease, is common. In a case of recurrence of IgA deposition in the graft, clinical presentation may vary from no symptoms [89] to hematuria, proteinuria, or even progressive kidney dysfunction [8]. In addition, IgA deposition may either occur alone or together with other significant renal pathologies, including chronic rejection [90,91]. Histologic or clinically significant IgAN recurrence varies in the published literature, specifically as a function of time after transplantation [90,91,92]. In the multicenter Post-Transplant Glomerular Disease (TANGO) study, the cumulative incidence of IgAN recurrence was 19 percent at 10 years and 23 percent at 15 years in the cohort of 504 kidney allograft recipients [93]. Moreover, in the same study, Uffing et al. [93] reported that the risk of graft loss was higher among patients with IgAN recurrence in comparison to those without recurrent IgAN (hazard ratio: 3.69 and 95% CI: 2.04–6.66). In the recent study, Napoletano et al. [94] found that patients with a more aggressive form of IgAN, who reached ESKD before 36 years of age, had a higher risk of recurrence after transplantation. In addition, they confirmed that recurrent IgAN, especially if clinically relevant, was associated with a worse graft outcome.

There is no evidence that mutations in complement factor H and the CFH-related genes are responsible for the increased risk of recurrence of IgAN in the graft. In addition, due to the lack of a prospective study involving protocol biopsies, the true risk of significant allograft dysfunction and/or allograft loss from IgAN recurrence disease is still unknown [95].

IgA deposition could be found in both deceased- and living-donor kidneys transplanted into non-IgAN recipients. Data in this regard is very limited. Ji et al. [96] studied the fate of the mesangial IgA deposits in the donor kidney after allograft transplantation (*n* = 83) in relation to the non-IgA deposition kidney group (*n* = 259). They found that glomerular mesangial IgA deposits gradually disappeared from the mesangial regions in grafts of acute rejection; however, the long-term outcome, i.e., allograft survival, was similar in both groups. Kidneys with known IgA deposits for donation should not be transplanted.

Historically, IgAV can recur in the graft in up to 60% of cases; however, it seldom causes clinical recurrence [97]. There are no significant differences between 10-year outcomes of kidney allograft recipients with IgAV relative to primary IgAN reaching 90% [98].

## 9. Limitations of GN Epidemiology Studies

The limitations of epidemiological studies in glomerulonephritis are substantial and multifactorial. Epidemiological data on glomerular diseases are mostly derived from biopsy-based registries or single-center series rather than truly population-based cohorts, which leads to strong selection bias. The indications for biopsy, such as nephrotic-range proteinuria or isolated hematuria, vary between countries and even centers. Patients who undergo a biopsy differ systematically by age, comorbidity, and access to care—some types of nephropathies are less frequently biopsied in specific populations, such as minimal change disease in the pediatric population or diabetic nephropathy in the elderly population. These differences in biopsy practices can shift the disease spectrum and may not reflect the true epidemiology.

Moreover, many low-income countries do not have the resources to provide complete diagnostics. Retrospective studies, with incomplete capture of data and limited follow-up, make accurate incidence, prevalence, and outcome estimates difficult and prone to information bias. Additionally, reliance on clinical coding (e.g., ICD codes) or claims data can lead to misclassification and incomplete capture of disease burden, as these methods may not distinguish between primary and secondary glomerulonephritis or accurately reflect histopathological subtypes.

Geographic disproportion in available data is impossible to ignore. High-income regions are heavily oversampled, while large portions of Africa, South Asia, and Latin America have sparse data, which often comes from centers treating the most severe, selected patient populations [29]. A Scottish study from an advanced healthcare system found that IgA nephropathy and deprivation are linked [99,100]. This suggests that socioeconomic deprivation is a risk marker worth investigating further.

Diagnostic reclassification over time complicates historical comparisons—splitting MPGN into IC-MPGN and C3G or recognizing PLA2R-positive MN complicates the direct comparison of historical and newer cohorts [101]. This may create artificial trends that reflect reclassification rather than true epidemiologic shifts [102,103].

Rare conditions such as C3G or ANCA-GN are typically reported in small case series with limited data on outcomes and prognostic factors [104,105]. International comparisons and temporal trends must be interpreted cautiously due to these limitations. Most epidemiological studies in GN lack standardized long-term follow-up and agreement on how to report outcomes, such as estimated glomerular filtration rate (eGFR), proteinuria, and histological features. This heterogeneity makes it nearly impossible to accurately characterize disease progression and prognosis across populations. In summary, underdiagnosis, selection bias, misclassification, regional disparities, and incomplete data reporting are key limitations in the epidemiological study of glomerulonephritis. Many of these limitations are most clearly illustrated by IgA nephropathy, where observed incidence, prognosis, and recurrence risk vary substantially according to biopsy practices and registry structure.

## 10. Translation into Clinical Practice

Knowledge of the epidemiology of glomerulonephritis directly impacts daily clinical practice by shaping the differential diagnosis, thereby guiding the selection of initial diagnostic tests and treatment strategies. For example, in the context of postinfectious glomerulonephritis, it is known that the condition most often occurs in children following throat or skin infections caused by group A β-hemolytic streptococci. However, its incidence has declined in high-income countries due to improved hygiene practices and public health measures [106]. Acute glomerulonephritis associated with non-streptococcal bacteria is increasingly observed among older, immunocompromised populations during ongoing chronic infection rather than in the postinfectious or latent phase. Moreover, the prognosis is favorable in children, while in the older group, in whom infection is associated with non-streptococcal pathogens, the risk of permanent renal dysfunction and the development of CKD is higher, which should dictate different management strategies by the medical team depending on the epidemiology [107]. Furthermore, knowledge of the epidemiology of the most common and severe CKDs provides important guidance for the development and selection of screening diagnostic tests. It allows for better allocation of human and financial resources and the development of the best public health strategies. We know that IgAN is significantly more common and often has a more aggressive course in the Asian population, while FSGS is diagnosed more frequently in the North American population, which therefore shapes the diagnostic approach for patients presenting with hematuria and proteinuria in these regions [108]. Furthermore, knowledge of the regional and age-related incidence of glomerulonephritis also allows for determining the likelihood of specific etiologies, which can lead not only to more optimal screening in primary care offices but also to targeted serological and histopathological evaluation [109]. Furthermore, epidemiology also shapes individual risk stratification and prognosis. Epidemiological reports often answer the most important patient questions regarding survival, quality of life, and risks related to reproductive plans. Some glomerulopathies, such as C3 glomerulopathies, have a variable prognosis depending on the underlying genetic abnormality or acquired complement pathway abnormalities, which are increasingly recognized in molecular epidemiology and influence the choice of treatment methods [110]. Identifying high-risk populations allows for optimization of monitoring and possible slowing of progression to stage G5 CKD [32,89]. Importantly, conclusions from epidemiological studies enable personalized therapy and the development of personalized medicine. The Kidney Disease: Improving Global Outcomes (KDIGO) guidelines emphasize that understanding the distribution, pathogenesis, and epidemiology of glomerular diseases enables the use of targeted therapies (e.g., B-cell depletion and complement inhibitors) and influences the selection of immunosuppressive treatment regimens, which reduces unnecessary exposure to immunosuppression and optimizes treatment outcomes [50,111].

Furthermore, epidemiological trends are driving changes in classification and treatment approaches. While the literature has historically been dominated by pattern-based classification of glomerulonephritis, there are now increasing publications suggesting a classification based on cause and immunopathogenesis, enabling more optimal treatment selection and better patient stratification in clinical trials [112]. Ultimately, epidemiological studies highlight the significant burden of undiagnosed or subclinical disease, particularly in populations with poor access to care due to age, gender, ethnicity, and geographic location, prompting efforts to address inequalities in access to healthcare and optimize resource allocation for prevention, diagnosis, and treatment [1,29]. Additionally, as it was shown in the recent KDIGO guidelines, interventions with reported efficacy in specific populations are based on epidemiology and trial results, i.e., mycophenolate mofetil and hydroxychloroquine in China and tonsillectomy in Japan [13].

Despite the tsunami of papers on IgA in recent years, the major wave deals with pathogenesis and novel therapies, including new randomized controlled trials together with systematic reviews and meta-analyses [113,114,115,116,117,118,119,120,121], including editorials such as the one recently published in the New England Journal of Medicine by Marcello Tonelli [122] or the review by Stoneman et al. [123] in JAMA. However, even with the most recent knowledge on pathogenesis, without proper and reliable epidemiology, designing a trial may bear the risk of under-recruitment, delayed recruitment, or the chance of not reaching the planned endpoints.

In addition, access to biopsy, including protocol biopsy (in particular, zero biopsy at the time of engraftment), immunofluorescence staining, and appropriate pathology assessment are crucial not only for the diagnosis but also for therapy, including how to cope with treatment failure. This is of utmost importance in transplantation to proceed with living donation and to deal with IgA recurrence in the graft, as well as when IgA is transferred with the donor kidney. Within this abundance of papers, data on epidemiology, despite the limitations of these studies, and the effect of gender, pregnancy, and transplantation in patients with IgAN on recurrent disease are scarce. Therefore, we focused on the comprehensive review, including these issues, and underlined the translation into clinical practice.

## 11. Conclusions

Viewed across epidemiological variability, diagnostic dependence, and prognostic heterogeneity, IgA nephropathy functions as a reference disease illustrating the structural challenges of primary glomerulonephritis research. A detailed analysis of previously published registries leads us to conclude that the epidemiology of GN is strongly influenced by biopsy practices and the availability of care. IgAN exhibits particularly wide geographic variability and clinical transformation, which has implications for everyday clinical practice and international recommendations. We currently know that, based solely on the experience of a single center, we cannot conclude the benefits and risks of GN therapy. New therapies and biomarkers require current population data and the involvement of numerous nephrology centers and societies worldwide. The lack of uniform, global, population-based registries remains the greatest barrier to everyday clinical practice. Data from epidemiological studies enable personalized therapy and the development of personalized medicine with more optimal treatment selection and better patient stratification in clinical trials. In the latest KDIGO guidelines, new treatment strategies for IgAN were discussed in detail, as well as a new goal of therapy: complete remission with a reduction in proteinuria to less than 0.3 g/d to prevent disease progression to end-stage kidney disease and kidney replacement therapy. Finally, there is still a significant burden of undiagnosed or subclinical diseases, particularly in low- and medium-income settings. Therefore, inequalities in access to healthcare and optimizing resource allocation for prevention, diagnosis, and treatment should be addressed with prompt efforts.

## Figures and Tables

**Table 1:** Epidemiology, temporal trend, and ESRD risk of various glomerulonephritis.

GN	MN	FSGS	MCD	ANCA-GN	MPGN/C3G
**Predominant age group**	Older adults	All ages	Children	Older adults	Children/mixed
**Incidence rate per 100,000/year**	1.2	0.8–1.7	0.2	1.0–1.4	0.1–0.2
**Geographic variation**	Moderate	High (APOL1-related)	Moderate	High (latitude-dependent)	High (infection-dependent)
**Temporal trend** **(last 40 years)**	Stable	Marked increase	Stable/slight decline	Increase	Decline (post-infectious)
**Leading** **region**	Europe	North America	Asia (pediatric)	Northern Europe/Oceania	South Asia/Middle East
**ESRD risk**	Moderate	High	Low (short-term)	High	Very high

GN: glomerulonephritis, MCD: minimal change disease, FSGS: focal and segmental glomerulosclerosis, MN: membranous glomerulopathy, MPGN/C3G: membranoproliferative glomerulonephritis/C3 glomerulopathy, and ANCA-GN: anti-neutrophil cytoplasmic antibody-associated glomerulonephritis.

**Table 2:** IgAN incidence rates and IgAN among biopsies in selected countries [6,9,11,35,36,37,38,39,40,41,42,43,44,45,46,47,48,49].

Region	Country	IgAN Incidence Rate (per 100,000/Year)	IgAN Among GN (% Biopsies)
Asia-Pacific	**Japan**	3.9–4.5	39.7–45
	**China**	3.8	45
	**Singapore**	3.2	42.2
	**South Korea**	3.5	–
	**Taiwan**	–	26
	**Thailand**	–	29.3
	**India**	–	20.5
	**Australia**	0.8	–
Europe	**Sweden**	2.9	35
	**United Kingdom**	2.1	–
	**Germany**	1.8	23
	**France**	1.6	–
	**Norway**	–	22.6
	**Denmark**	1.31	–
	**Netherlands**	0.76	–
	**Romania**	–	13.9
North America	**United States**	1.3–2.2	11.8
	**Canada**	1.2	–
Latin America	**Mexico**	1.1	–
	**Brazil**	0.8	17.4
	**Colombia**	0.7	–
Sub-Saharan Africa	**Kenya**	0.2	–
	**Nigeria**	0.15	–
	**South Africa**	0.05	–

**Table 3:** Epidemiology, pathogenic mechanism, and treatment of primary glomerulonephritis.

GN	IgAN	MN	FSGS	MCD	ANCA-GN	MPGN/C3G
**Global Incidence rate per 100,000/year**	2.5	1.2	0.8–1.7	0.2–0.6	1.0–1.4	0.1–0.2
**Predominant Geographic Region**	East Asia	Europe	North America	Asia	Northern Europe Oceania	South Asia Middle East
**Key Pathogenic mechanism**	Aberrant IgA O-galactosylation. Mesangial deposition with complement activation.	Autoimmune targeting podocytes: PLA2R, THSD7A. Classical complement pathway activation.	Multiple pathways: genetic, APOL1-associated, acquired.	Podocytes dysfunction with loss of charge selectivity.	ANCA-neutrophil activation. Necrotizing vascular inflammation. Crescent formation.	Immune complex or/and complement deposits. Thickening of capillary walls and mesangial proliferation.
**Typical Clinical Syndrome**	Asymptomatic hematuria, infrequently nephrotic syndrome	Nephrotic syndrome	Nephrotic syndrome	Nephrotic syndrome	RPGN, systemic vasculitis	RPGN, rapid decline of kidney function
**First-line therapy**	ACE-I/ARB and SGLT-2 targeting proteinuria, oral budesonide, new targeted therapies–iptacopan, narsolimab	High-risk: CYC + CS or RTX Low-risk: ACE-I/ARB, anticoagulation	ACE-I/ARB, CS Essential genetic testing	First line: CS Second line: CNI, MMF, RTX	Urgent induction: RTX or CYC, CS. Emerging: avacopan	Similar to ANCA-GN New complement inhibitors: iptacopan, pegcetacoplan
**Biomarker**	Genetic testing, no-specific serum marker	Anti-PLA2R	APOL-1, genetic testing	Non-specific	ANCA MPO or PR3	Immunocomplex and/or complement deposits

GN: glomerulonephritis, IgAN: IgA nephropathy, MCD: minimal change disease, FSGS: focal and segmental glomerulosclerosis, MN: membranous glomerulopathy, ANCA-GN: anti-neutrophil cytoplasmic antibody-associated glomerulonephritis, MPGN/C3G: membranoproliferative glomerulonephritis/C3 glomerulopathy, RPGN: rapidly progressive glomerulonephritis, ACE-I: angiotensin-converting enzyme inhibitor, ARB: angiotensin receptor blocker, SGLT-2: sodium–glucose co-transporter 2, CYC: cyclophosphamide, CS: corticosteroid, RTX: rituximab, and CNI: calcineurin inhibitor.

## Data Availability

No new data were generated.

## Supplementary Materials

The following supporting information can be downloaded at https://www.mdpi.com/article/10.3390/jcm15052046/s1, Table S1: Preferred Reporting Items for Systematic reviews and Meta-Analyses extension for Scoping Reviews (PRISMA-ScR).

## Abbreviations

The following abbreviations are used in this manuscript:

AAV	ANCA-associated vasculitis
ANCA	anti-neutrophil cytoplasmic antibody
C3G	C3 glomerulopathy
eGFR	estimated glomerular filtration rate
ESKD	end-stage renal disease
FSGS	focal segmental glomerulosclerosis
GBD	Global Burden of Diseases, Injuries, and Risk Factors
GN	glomerulonephritis
IC-MPGN	Immune-Complex Membranoproliferative Glomerulonephritis
INS	Idiopathic Nephrotic Syndrome
IgAN	IgA nephropathy
KDIGO	the Kidney Disease: Improving Global Outcomes
MEST-C	Mesangial hypercellularity, Endocapillary hypercellularity, Segmental glomerulosclerosis, Tubular atrophy/interstitial fibrosis, and Crescents
MN	Membranous nephropathy
MPGN	membranoproliferative glomerulonephritis
PLA2R	Phospholipase 2 receptor
WHO	World Health Organization
